# Sports chiropractic management at the World Ice Hockey Championships

**DOI:** 10.1186/1746-1340-18-32

**Published:** 2010-12-03

**Authors:** Chris Julian, Wayne Hoskins, Andrew L Vitiello

**Affiliations:** 1 Queenstown Health, 38B Gorge Rd, Queenstown 9300, New Zealand; 2 Department of Surgery, Royal Melbourne Hospital, Grattan St, Parkville 3050, Victoria, Australia; 3 Department of Academic Affairs, Anglo-European College of Chiroparctic, 13-15 Parkwood Road, Bournemouth BH5 2DF, UK

## Abstract

**Background:**

Ice hockey is an international sport. Injuries occur in a full body fashion, to a number of tissues, commonly through body contact. There is a lack of literature documenting the scope of sports chiropractic practice. Thus, it was the aim to document the type, scope and severity of conditions presenting to, and the treatment provided by, the New Zealand team chiropractor acting as a primary health provider for the duration of the 2007 World Ice Hockey Championships.

**Methods:**

All conditions presenting were recorded. Diagnosis was recorded along with clinical parameters of injury: injury type, severity, mechanism and whether referral or advanced imaging was required. All treatment provided was continuously recorded, including information on the number of treatments required and the reason, duration, type and location of treatment.

**Results:**

Players presented for diagnosis of injury 50 times. Muscle (34%), joint (24%) and tendon injuries (18%) were most common. Players presented with a new injury 76% of the time. Most injuries had been present for less than one week (84%), with 53% occurring through a contact mechanism. Injuries were common at training and match locations. Only two injuries required the player to stop playing or training, both of which were referred for advanced imaging. During the study, 134 treatment consultations were rendered to 45 player injuries. Eighty per-cent of injuries were managed with four or less treatments. Three quarters of treatment was provided at training locations with treatment duration predominantly being between 11-15 minutes (71%) and 16-20 minutes (27%). Most treatment delivered was passive in nature (71%) although combination active and passive care was provided (27%). Treatment typically involved joint (81%) and soft tissue based therapies (81%) and was delivered in a full body manner.

**Conclusions:**

This study documented the injury profile of ice hockey at an international level of competition. It documented the conditions presenting to a chiropractor for diagnosis and the treatment provided. Treatment was consistent with that recommended for chiropractic management of athletic injuries. This documentation of sports chiropractic scope of practice fills a void in the literature and assists in determining a role for sports chiropractors as primary health providers or in multidisciplinary sports management teams.

## Background

Ice hockey is a body contact sport played through North America, Europe, Russia and other parts of the world. Teams consist of five players on the ice at any one time in addition to a goal-keeper and up to 15 on an interchange bench. Each game is played over three 20 minute periods plus stoppage time. Body contact plays a significant role in this power sport, with collisions producing a significant number of injuries [1], such that body checking and unintentional collision with an opponent are the most common mechanisms of injury [2]. Forwards have the highest rate of injury, followed by defensemen and then goalkeepers [3,4]. The rate of injury has been found to be more than eight times higher in games than in practices where physical collisions do not occur to the same frequency or intensity [5]. Injuries can and do frequently occur to the lower extremity, pelvis and hip [5], head, neck and face [4]. Contusions are the most common form of injury, followed by strains, lacerations, and sprains [3]. Despite the body contact nature of the game, players are prone to sprains and strains, which may not involve any body contact [4]. However, it should be noted that injury rates and risks are potentially different at different levels of play, between men and women and in different countries. The full body injury profile of predominant acute onset injury represents a challenge for the sports clinician in terms of diagnosis and management.

There is a lack of literature documenting the scope of chiropractic practice in the sport setting of ice hockey for this calibre of play. In particular there is a lack of published recording of the conditions presenting to chiropractors and the chiropractic management provided to athletes at sporting events or in private practice [6], whereas other professions have documented this [7-10]. This lack of literature has contributed to difficulties in defining sports chiropractic and identifying how sports chiropractors differ, if they do, from general chiropractors and physiotherapists [11]. This may be a contributing factor in the difficulty sports chiropractors face in securing positions in many team sports and sporting organizations [12]. An increased amount of scientific literature documenting the conditions that sports chiropractors treat and the management they provide may help guide any future recognition for the profession as a whole.

Considering that chiropractors are capable of providing a full body treatment approach [6,13,14], it would seem that sports chiropractors would be suited to the injury management demands occurring in the sport of ice hockey. Thus it was the aim of this research to document the type, scope and severity of conditions presenting to the New Zealand team chiropractor for the duration of the 2007 World Ice Hockey Championships. Additionally, it was the aim to systematically document the scope of sports chiropractic treatment provided by the chiropractor. This information would give an idea of the injury profile of ice hockey and document the true scope of management of a sports chiropractor.

## Methods

The study was conducted for the duration of the 2007 World Ice Hockey Championships DivIII held in Dundalk, Ireland. The duration of the study included the pre-event tour and training camp for the male New Zealand team and the period of competition match play; total time span four weeks. All players from the New Zealand male ice hockey squad were recruited as subjects for this study. The team chiropractor was the sole primary health provider for the team whose role was to perform diagnostic triage to refer out red flag conditions and to diagnose and treat injuries amenable to chiropractic care. The chiropractor was the sole primary health provider as due to a limited budget a larger, multidisciplinary medical team was not possible even though it may have been preferable. The team did have very limited access to additional massage services. It is not usual occurrence for chiropractors to be the sole medical provider at such an event or level of competition. Treatment and management was delivered within the rules governing chiropractic in New Zealand [15,16] and in accordance with the Accident Compensation Corporation (ACC) treatment guidelines [17]. The study conformed to the ethical standards and requirements of the Anglo-European College of Chiropractic (AECC) Research Ethics Sub-Committee, *who determined that ethical approval was not required*.

### Initial consultation/new injury

**Figure 1:**
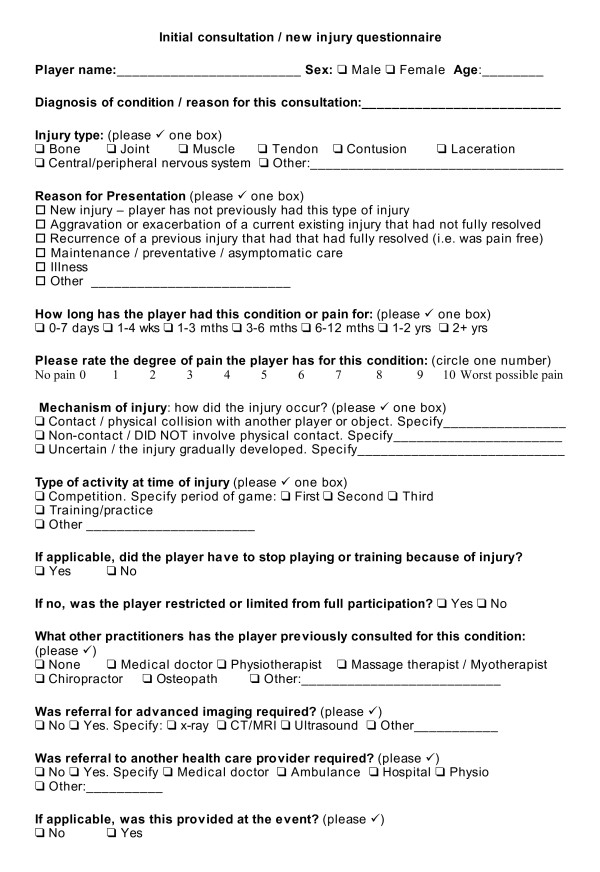
**Initial consultation/new injury questionnaire**.

For all players presenting for injury diagnosis at initial consultation a form presented in Figure 1 was filled in. The questionnaire was developed by the study investigators and thoroughly pilot tested in private practice, with minor corrections made during the testing process. Information was recorded on player characteristics as well as clinical parameters of injury such as diagnosis, injury type, reason for presentation, duration of injury, severity, mechanism of injury as well as information on previous treatment and imaging. Injury recording was based on the Orchard Sports Injury Classification System (OSICS) [18]. The OSICS system was chosen because it is a freely available, encompassing system with moderate levels of inter-rater reliability for recording sports injuries. Injury severity was measured using a visual analogue scale (VAS). The remainder of the questionnaire was designed because the focus of the study is something that no other investigators have targeted in clinical surveys, with specific questions asked which are not covered in other questionnaires. Information on player anthropometrics and playing experience was not recorded as this was not the focus of the study. Individual breakdown of exact playing and training time and playing position was also not recorded.

### Treatment

**Figure 2:**
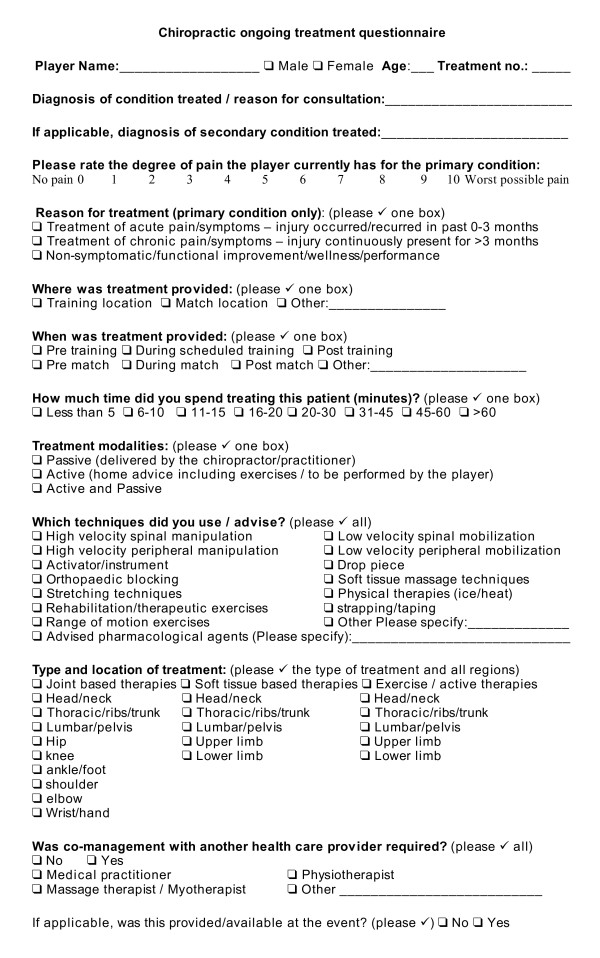
**Chiropractic ongoing treatment questionnaire**.

All management rendered to the players was continuously recorded using the form presented in Figure 2. The questionnaire was developed by the study investigators and thoroughly pilot tested in private practice, with minor corrections made during the testing process. Information was recorded on the number of treatments for each player injury, the diagnosis of injury, severity of symptoms, reason for treatment, where and when treatment was provided, the duration of treatment, treatment modalities used, the type and location of treatment and whether co-management was required. For severity of injury, players completed the VAS at diagnosis or prior to each treatment. The definition of injury was that presenting for diagnosis. From here the injury was managed which may have required a number of treatment sessions. If pain was rated zero by the player/patient and some functional deficit was still present (e.g. decreased range of motion, loss of strength etc), management may have continued to address this.

## Results

There were 22 players in the New Zealand squad (age range 17-31 y, mean 22.5 y). The team played three pre-tournament matches and five tournament matches and had 16 training sessions with length or time varying between 60-90 minutes.

### Initial consultation/new injury

The average age of players presenting with injury was 22.7 y (range 18-30 y). Players presented for diagnosis of injury 50 times throughout the course of the study with the body regions and diagnoses provided in Table 1. Injuries occurred to 19 out of the 22 players. The most common injuries were muscle injuries (34%), joint injuries (24%), tendon injuries (18%) and contusions (6%). Medical illnesses (all symptoms consistent with acute viral gastroenteritis which fully resolved in 24-48 hours) provided 10% of initial consultation (which are not presented here). Players presented with a new injury 76% of the time, a recurrence of a resolved injury 13% and aggravation or exacerbation of a current existing injury 7%. At the time of diagnosis, most injuries had been present for less than one week (84%), followed by one-to-four weeks (10%), one-to-three months (4%) and three-to-six months (2%). Regarding severity of injuries the mean on a visual analogue scale (VAS) was 4.1 (range 0-8, SD 1.8). Most injuries occurred through a contact mechanism (53%), with non-contact (31%) and unsure or gradual onset (16%) less likely. Injuries occurred through a mix of match and training with 49% of injuries occurring during training, 40% during matches and 11% during other activities or unsure onset. For the match injuries the bulk occurred during the second period of play (56%), with less during the third (33%) and first periods (6%). Only two injuries required the player to stop playing or training, suggesting that players were prepared to carry discomfort given the level of pain indicated by the results of the VAS. Two players were referred for imaging, with plain film X-rays performed: one of these players was referred to a general medical practitioner first who subsequently requested imaging, and one directly to hospital for further investigation.

**Table 1:** Diagnosis breakdown of initial consultations for new injuries

Body region	Number (%)	Details of diagnosis
Head/neck	7 (14%)	7 neck sprain/strains

Shoulder/arm/elbow	7 (14%)	6 shoulder sprains/dislocations, 1 sternoclavicular joint sprain

Forearm/wrist/hand	3 (6%)	1 fracture, flexor digitorum tendinosis, 1 finger haematoma

Trunk/spine	10 (20%)	8 lumbar/thoracic sprain/strains, 2 thoracic spine haemtomas

Hip/groin/thigh	9 (18%)	4 groin strains, 1 hamstring strain, 3 thigh haematomas, 1 gluteus medius/tensor fascia latae strain

Knee	2 (4%)	1 knee cartilage injury, 1 patellar tendon injury

Shin/ankle/foot	7 (14%)	1 ankle sprain, 2 calf strains, 2 tibialis posterior tendinosis, 1 tibialis anterior tendinosis, 1 foot haematoma

Medical illness	5 (10%)	Symptoms consistent with gastroenteritis

**Total injuries**	50	

### Treatment

**Figure 3:**
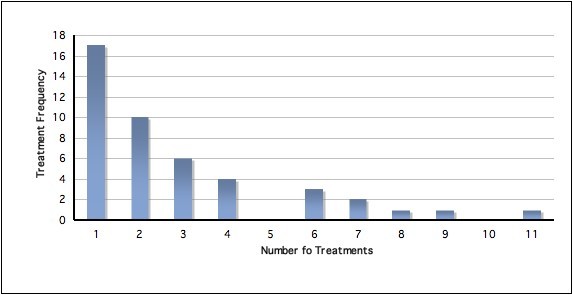
**Number of treatment consultations provided**.

During the course of the study, 134 treatment consultations were rendered to 45 player injuries (mean 2.98 consultations per injury, SD 2.5) with further details presented in Figure 3. Treatment was largely short term in nature with 36% of player injuries requiring one treatment and 80% four or less treatments. The mean severity of pain experienced at treatment sessions was 2.9 (range 0-8, SD 2.0). Regarding the reason for treatment, 86% was primarily for the management of acute pain/symptoms with 13% for non-symptomatic or functional improvement. Treatment was mostly provided at training locations (75%) and less at matches (25%), with treatment almost exclusively provided either before training (23%) or matches (22%), or after training (48%). Very little treatment was provided during training or during matches, or after matches. Duration of treatment was predominantly 11-15 minutes (71%) but also 16-20 minutes (27%) or six-to-ten minutes (13%). Only 3% was five minutes or less. Most treatment delivered was passive (delivered by the chiropractor) in nature (71%), although combination active and passive care was provided (27%) with very little active (performed by the patient independent of the chiropractor) only treatment (2%), results which likely represent the acute nature of most injuries. Table 2 presents the results of the treatment techniques provided which reflected a multimodal treatment paradigm. This typically consisted of high-velocity low-amplitude (HVLA) spinal manipulation, soft tissue massage techniques, extremity mobilisations and manipulation along with rehabilitation/strengthening and stretching techniques. Treatment typically involved joint and soft tissue based therapies with 81% of all treatment consultations involving joint based therapies, 81% soft tissue therapies and 25% exercise based or active therapy. For joint based therapies treatment was delivered in a full body manner to the spine and extremities, but largely to the thoracic spine (34%), lumbar/pelvis (28%) and neck (20%). Soft tissue therapies were also delivered in a full body manner with 33% to the lumbar/pelvis, 25% to the lower limb, 15% to the thoracic area, 14% to the upper limb and 13% to the head/neck. Exercise based active therapies largely involved the lower limb (44%), upper limb (26%) and lumbar/pelvis (15%). Co-management was rarely required with only one consultation requiring medical assistance and four consultations requiring additional massage assistance.

**Table 2:** Treatment techniques provided for the 134 treatment consultations

Treatment technique	Number
High-velocity, low-amplitude spinal manipulation	100

Spinal mobilisations	0

Extremity high-velocity, low-amplitude manipulation	19

Extremity mobilisations	39

Instrument assisted	0

Drop piece	1

Orthopaedic blocks	0

Soft tissue massage techniques	107

Stretching techniques	20

Physical therapies (heat/ice)	13

Rehabilitation exercises	24

Strapping	6

ROM exercise	5

Medication/pharmaceutical advice	3

## Discussion

The results of this study showed that less severe injuries, requiring treatment but not missed competition or training, commonly occur in ice hockey with 19 of 22 players presenting for chiropractic care at least once. Injury occurred in a full body distribution, occurring most commonly to the lower extremity (40%), trunk/spine (22%), upper extremity (22%) and head/neck (16%). The most common conditions presenting for treatment involved muscle, joint and tendon injuries. By far the majority of injuries were acute and new onset, occurring through a blunt contact mechanism. Injuries occurred commonly during match and training sessions. Treatment of injuries provided by the chiropractor in this study was multimodal in nature. It consisted of a full body approach with mainly passive therapies although active therapies were also provided. Treatment was delivered to both joint and soft tissues equally and treatment typically incorporated HVLA spinal manipulation, soft tissue massage techniques, extremity mobilizations along with rehabilitation/strengthening and stretching techniques. Four or less treatments were required to treat most injuries, with treatment provided at predominantly training locations. Treatment lasted approximately 15 minutes on average.

The injury surveillance results in this study were similar to other results published in the scientific literature [2-5], although this study demonstrated more injuries occurred during training whereas other literature suggests most injuries occurred during matches [3,4]. Future research is required to identify why there was such a high amount of training injuries occurring with training or coaching methods possibly contributing, with opportunities for prevention of injury possible. Injuries occur most commonly during games as a result of collisions [19], with player-to-player contact the mechanism of half of all match injuries in one study [5]. The reason for a high amount of training injuries in this study could be because the pre-tournament training camp was included where matches were not being played so training scenarios were close to game situations, and other studies are likely to have been conducted during domestic seasons where heavy playing schedules (three times per week in some cases) generally mean less body contact based training scenarios. A high prevalence of concussion is known to occur in ice hockey [4], although these injuries did not feature in our study. It should be noted that chiropractors are qualified to diagnose concussion and to provide first aid management and this is covered in undergraduate training [11]. The low rate of concussion could be because international ice hockey is played on a larger ice surface compared to most professional leagues, reducing likelihood of collisions and it also has stricter rules on body contact and fighting, ensuring a reduction in the chance of head injury. Similar to the literature we also found lower extremity injuries to be the most prevalent [5], although internal knee derangements feature more prominently in other studies [5]. The rates of knee joint injury in ice hockey has caused concern in the literature [4]. As most injuries in our study occurred during the second period of play, this suggests that lack of warm up and fatigue were not the primary contributors of injury. This makes identification of risk factors for these injuries and subsequent prevention perhaps more difficult.

Despite the high amount of body contact in ice hockey and supporting our findings that muscle injuries were the most common injury to occur, non-contact injuries frequently occur with sprains and strains accounting for 40% of injuries in one study [4]. Muscle strains of the pelvis and hip muscles have been documented to be the most common injury reported during training in one study [5]. Given the non-contact nature of these injuries, this suggests prevention of these injuries may by achievable and identification of risk factors is required. Similar to the evidence present in the literature, our study also found a high percentage of injuries requiring only short-term treatment, with most injuries requiring less than seven days to return to full activity in one study [4].

The treatment provided in this study reflected the full body incidence of injury in ice hockey. It has been discussed that sports chiropractors need an expert knowledge of injury epidemiology and injury mechanisms experienced in the chosen sport of the athletic patient, along with information regarding risk factors for injury, etiological factors, biomechanics and anatomy [11]. The treatment provided was representative of the "modern" multi-modal (MMM) chiropractic approach [6]. The MMM approach used by sports chiropractors is said to incorporate components of passive and active care to address both the acute inflammatory/pain phase and the chronic/rehabilitation/injury prevention phase of injury [11]. The full body treatment approach incorporating passive and active techniques would seem to be quite different from care provided by general practitioner chiropractors [11], although a lack or similar research documenting the scope of practice of general chiropractors makes comparisons difficult.

Also limited is literature documenting the scope of practice of other professions in sports medicine, in particular sports physiotherapy. Research conducted at the Olympic polyclinic on the management provided by 73 experienced physiotherapists shows similar results to this study, in that the mean treatment sessions provided was 4.4 (range 1-44) [9]. The majority of patients (54%) had fewer than three sessions, and only 6% had more than 10 sessions. However, the treatment modalities differed to our study, where modalities most commonly used were ultrasound, massage, manual therapy techniques, therapeutic exercise, cryotherapy, taping and transcutaneous electrical nerve stimulation (TENS). A breakdown of the type of the specific manual therapy technique is not specified. Similar literature from the Pan-American Games has also been performed [10]. The most common modalities used were kinesiotherapy (defined as muscle strengthening and/or flexibility exercises) (24.9% of all total treatments), ultrasound (19.4%), cryotherapy (17.2%), superficial heat (12.8%), interferential current (11.1%), TENS (7.3%), with osteopathy rarely used (0.6%). This corresponded to an average of 1.54 procedures per treatment consultation, suggesting closer to a unimodal style of practice, not multimodal as was performed in this study. Based on this limited literature available from both professions, it would suggest the treatment techniques, modalities and style of practice differ between sports chiropractors and sports physiotherapists, with manual therapies and HVLA manipulation being more prominent in chiropractic [20]. However, comparative research is required to further assess this. Research should also further investigate the benefits of HVLA manipulation in sporting populations given its possible role in injury prevention [21] and performance enhancement [22]. Furthermore, it should be noted that because of a lack of funding, the team in our study did not have a travelling masseur or physiotherapist or one available for the majority of the time. Multidisciplinary management would have been appropriate in the management of many cases if it had been available. Multidisciplinary co-management may have produced a difference in the results of this study and this change in treatment should be further investigated in future studies.

A recent published paper has highlighted the key criteria and principles that are thought to be important in the identification of an appropriate chiropractor for the management of athletic injuries [23]. The treatment provided in our study fitted these criteria, with treatment being of sufficient treatment time, multimodal in nature, containing active and passive components, not requiring mandatory x-rays or predetermined schedules of care. Medical terminology was also used and diagnosis provided. The results of our study support the further use of these criteria when selecting a chiropractor for the management of athletic injuries. Given the full body nature of injuries occurring in ice hockey and other sports, it suggests that some chiropractors are not suited to the management of these athletes [24], particularly chiropractors with a unimodal therapy approach (i.e. manipulation only and often in one single style) [11]. These unimodal practitioners are often thought to be representative of the sports chiropractor [12], however available evidence suggests this is not the case.

As far as we are aware, this is the most detailed study of its type providing continuous recording of all diagnoses and treatment rendered to document the scope of practice in sports chiropractic. The study should be expanded as a clinical practice survey and implemented in multi-centre studies to provide an accurate representation of sports chiropractors. Future study could use chiropractors managing athletes from a range of sports and from private practice. Future, larger research projects could also consider reporting the number of each new injury as well as the percentage of total new injuries and repeat injuries that this represents, as this study was not large enough to warrant analysis of repeat injuries. Similar research recruiting general chiropractors and other sub-specialties of chiropractic should also be performed to present definitive data on the scope of chiropractic practice and to provide a clear delineation between the subdivision of the various subtypes of chiropractors which exist. Future research is encouraged to also include data on adverse events that may or may not occur from treatment, such that an accurate benefit: risk ratio can be documented. The accumulation of data in multi-centre studies could allow publication of large case series, which would be capable of documenting the number of treatments required for management before discharge. Randomised controlled trials should be performed to investigate effectiveness of treatment using the VAS and other functional outcome measures. Such study is pertinent given the lack of chiropractic literature on management of extremity conditions in particular [6,13,14].

Limitations exist in the study conducted. Firstly, there are limitations in the generalisability of this study as it is a small study performed on only one team by an individual chiropractor. A larger study, performed over a longer period using multiple teams and chiropractors would give more accurate data. Also, it is possible that the number of injuries is underestimated as some players may have elected not to receive diagnosis and treatment for their condition. If this occurred it would more likely be for more minor and self-limiting conditions. Any injury definition has a threshold limit, but it is less likely that more severe injuries were missed as such injuries have greater reliability in reporting [25]. Information on the number of treatments may be an underestimate given an endpoint existed in the study and some injuries may not have resolved and would have required further treatment. Further treatment may also have required different treatment strategies than which were presented in this study, such as increased therapeutic exercise and rehabilitation to prevent chronic and recurrent injury. When considering the duration of treatment, it needs to be considered that most treatment was provided before training and matches, and a time constraint existed. In an ideal situation or with a larger management team, a longer duration of treatment may have been provided.

## Conclusions

This study documented the profile of injuries occurring in the sport of ice hockey. It demonstrated that a sports chiropractor for the New Zealand ice hockey team when acting as the primary health provider was required to diagnose conditions occurring in a full body distribution and to a number of tissue types. Diagnostic triage was performed with referral of conditions not amenable to chiropractic management. Treatment provided was multimodal and full body in nature. It consisted of joint, soft tissue and active therapies. Most injuries were managed through a short course of treatment with the duration of treatment consistent with that recommended in the literature. Given the documentation of the sports chiropractic scope of practice and management strategies it may delineate a role for sports chiropractors as primary health providers or as part of a multidisciplinary management team, which would provide best practices for the injury management of athletes. Further research is required to expand on the differences that appear to exist between the scope of practice of sports chiropractors and general chiropractors and physiotherapists, and whether this produces different clinical outcomes.

## Competing interests

Potential conflict of interest may exist in reporting this study as the paper promotes the use of chiropractors in sports medical teams. No source of funding was used in the preparation of this manuscript.

## Authors' contributions

CJ, WH and AV conceived the idea of the study and formulated the aims and methodology. WH designed the questionnaires. CJ provided and recorded all diagnoses and treatment. AV sought ethics approval for the study. All authors contributed to writing the multiple drafts and the final document. All authors read and approved the final document.
